# Alcohol and Mortality: Global Alcohol-Attributable Deaths From Cancer, Liver Cirrhosis, and Injury in 2010

**DOI:** 10.35946/arcr.v35.2.07

**Published:** 2014

**Authors:** Jürgen Rehm, Kevin D. Shield

**Affiliations:** **Jürgen Rehm, Ph.D.,***is Director, Social and Epidemiological Research (SER) Department, Centre for Addiction and Mental Health, Toronto, Canada; Professor, Institute of Medical Science, University of Toronto, Canada; Chair Addiction Policy, Dalla Lana School of Public Health, University of Toronto, Canada; Professor, Department of Psychiatry, University of Toronto, Canada; Head, PAHO/WHO Collaborating Centre for Mental Health & Addiction; and Head, Technische Universität Dresden, Klinische Psychologie & Psychotherapie, Dresden, Germany.*; **Kevin D. Shield, MH.Sc.,***is a pre-doctoral fellow, Social and Epidemiological Research (SER) Department, Centre for Addiction and Mental Health, Toronto, Canada, and Ph.D. student at the Institute of Medical Science, University of Toronto, Canada.*

**Keywords:** Alcohol consumption, alcohol burden, alcohol-attributable mortality, alcohol-attributable fractions, global alcohol-attributable mortality, risk factor, cancer, liver cirrhosis, injury, burden of disease, Global Burden of Disease and Injury study

## Abstract

Alcohol consumption has long been recognized as a risk factor for mortality. By combining data on alcohol per capita consumption, alcohol-drinking status and alcohol-drinking patterns, risk relationships, and mortality, the Comparative Risk assessment study estimated alcohol-attributable mortality for 1990 and 2010. Alcohol-attributable cancer, liver cirrhosis, and injury were responsible for the majority of the burden of alcohol-attributable mortality in 1990 and 2010. In 2010, alcohol-attributable cancer, liver cirrhosis, and injury caused 1,500,000 deaths (319,500 deaths among women and 1,180,500 deaths among men) and 51,898,400 potential years of life lost (PYLL) (9,214,300 PYLL among women and 42,684,100 PYLL among men). This represents 2.8 percent (1.3 percent for women and 4.1 percent for men) of all deaths and 3.0 percent (1.3 percent for women and 4.3 percent for men) of all PYLL in 2010. The absolute mortality burden of alcohol-attributable cancer, liver cirrhosis, and injury increased from 1990 to 2010 for both genders. In addition, the rates of deaths and PYLL per 100,000 people from alcohol-attributable cancer, liver cirrhosis, and injury increased from 1990 to 2010 (with the exception of liver cirrhosis rates for women). Results of this paper indicate that alcohol is a significant and increasing risk factor for the global burden of mortality.

## Alcohol and Mortality

Alcohol is causally linked to more than 200 different diseases, conditions, and injuries (as specified in the *International Classification of Diseases, Revision 10* [ICD-10] three-digit codes [see Rehm 2011; Rehm et al. 2009; Shield et al., 2013*c* [pp. 155–173 of this issue]). All of these disease, condition, and injury categories cause mortality and disability, and, thus, alcohol consumption causes a net burden of mortality and disability (Rothman et al. 2008). However, certain patterns of alcohol consumption are protective for ischemic diseases (Roerecke and Rehm 2012*a*) and diabetes (Baliunas et al. 2009), and, thus, alcohol can prevent death and disability from these causes. The total mortality and disability caused by and prevented by the consumption of alcohol is calculated by comparing the expected mortality under current conditions to a counterfactual scenario where no one has consumed alcohol (Ezzati et al. 2006; Walter 1976). Although the counterfactual scenario seems unrealistic as almost one-half of the global population consumes alcohol (for the most up-to-date statistics on alcohol consumption, see Shield et al. 2013*b*; World Health Organization 2011*a*), recent natural experiments in countries where there has been a considerable reduction in alcohol consumption showed a clear reduction in mortality (e.g., Russia) (Leon et al. 1997; Neufeld and Rehm 2013). Accordingly, the calculations of the deaths and disability caused by alcohol consumption seem to correspond to real phenomena and, thus, could predict an approximate level of reduction in mortality if alcohol consumption were to be reduced.

This article outlines the alcohol-attributable mortality burden from three major causes: cancer, liver cirrhosis, and injury. All three categories have long been identified as causally linked to alcohol consumption. With respect to cancer, in 1988 the International Agency for Research on Cancer established alcohol as a carcinogen (International Agency for Research on Cancer 1988), and in its latest monograph has found alcohol consumption to be causally associated with oral cavity, pharynx, larynx, esophagus, liver, colon, rectum, and female breast cancers (International Agency for Research on Cancer 2010, 2012). Studies have shown that stomach cancer may be associated with alcohol consumption, but evidence on the causal relationship between stomach cancer and alcohol consumption is not yet conclusive (International Agency for Research on Cancer 2012; Rehm and Shield, in press). Biologically, it has been established that ethanol, and not other ingredients of alcoholic beverages, is the ingredient that mainly causes cancer (Lachenmeier et al. 2012), with acetaldehyde (the first metabolite of ethanol) likely being the most important biological carcinogen (International Agency for Research on Cancer 2010, 2012; Rehm and Shield, in press). In addition, observational studies have found a clear dose-response relationship between alcohol consumption and the risk of cancer, with no observed threshold for the effect of alcohol, as an elevated risk of cancer has been observed even for people who consume relatively low amounts of alcohol (Bagnardi et al. 2013; Rehm et al. 2010*a*).

Liver cirrhosis has been associated with alcohol consumption, especially heavy consumption, since the seminal work of Benjamin Rush (Rush 1785). The causal link between alcohol consumption and liver cirrhosis is so strong and important that the World Health Organization has created a specific category for alcoholic liver cirrhosis (World Health Organization 2007). As with cancer, there is a dose-response relationship between alcohol consumption and the risk of liver cirrhosis, with no lower threshold being observed (Rehm et al. 2010*c*); however, the majority of the effect can be seen for heavy drinking (Rehm et al. 2010*c*).

The risk of injury also has been causally linked to alcohol consumption, with this relationship fulfilling all of the classic Bradford Hill criteria (e.g., consistency of the effect, temporality, a dose-repsonse relationship with the risk of an injury [biological gradient]) (Rehm et al. 2003*a*). The effect of alcohol on injury is acute; the level of risk for both intentional and unintentional injuries is clearly linked to blood alcohol level (Taylor and Rehm 2012; Taylor et al. 2010), with a very low threshold (Eckardt et al. 1998). There also is an association between average consumption of alcohol and injury (Corrao et al. 2004).

Alcohol-attributable cancer, liver cirrhosis, and injury constitute the majority of the burden of alcohol-attributable mortality. Collectively, they were responsible for 89 percent of the net burden of alcohol-attributable mortality (i.e., the mortality rate after including the beneficial effects of alcohol on ischemic diseases and diabetes) and for 79 percent of the gross burden of alcohol-attributable mortality (Shield et al. 2013*a*) in the United States in 2005, for people 15 to 64 years of age. Additionally, they were responsible for 91 percent of the net alcohol-attributable mortality and 79 percent of the gross alcohol-attributable mortality in the European Union (Rehm et al. 2012) and 80 percent of the net alcohol-attributable mortality and 72 percent of the gross alcohol-attributable mortality globally (Rehm et al. 2009) in 2004. This article does not review the other causes of alcohol-attributable deaths included in the latest Comparative Risk Assessment (CRA) Study (Lim et al. 2012). The CRA study estimates as published in December contained significant errors in the calculation of alcohol-attributable cardiovascular deaths, estimating that 33 percent of all ischemic heart disease deaths were attributed to alcohol, which is an impossibility as the relationship between alcohol consumption and this disease category is mainly protective (for details on relationship between alcohol and heart disease, see Roerecke and Rehm 2010, 2012*b*). A comparison with other alcohol-attributable disease and protective effects will thus only be possible once the corrected CRA results are published.

## Methodology Underlying the Estimation of the Mortality Burden of Alcohol-Attributable Diseases and Injuries

The number of alcohol-attributable cancer, liver cirrhosis, and injury deaths in 1990 and 2010 were estimated using alcohol-attributable fractions (AAFs) (Benichou 2001; Walter 1976, 1980). AAFs are calculated by comparing the population risk of a disease under current conditions to a counterfactual scenario where no one has consumed alcohol. This is achieved by using information on the distribution of levels of alcohol consumption and the associated relative risks (RRs) (i.e., risks of disease for different levels of alcohol consumption versus abstainers). These calculated AAFs then were applied to mortality data obtained from the 2010 Global Burden of Disease (GBD) Study for 1990 and 2010 (Lim et al. 2012). Mortality data for 1990 and 2010 were modelled using data on mortality from 1980 to 2010. Data on mortality were imputed for those countries with little or no data by using data from other countries and were smoothed over time (in addition to other data corrections procedures that corrected for cause of death recording errors) (Lozano et al. 2012).

### Calculating the Alcohol-Attributable Mortality Burden of Cancer and Liver Cirrhosis

Alcohol consumption is causally related to mouth and oropharynx cancers (ICD-10 codes: C00 to C14), esophageal cancer (C15), liver cancer (C22), laryngeal cancer (C32), breast cancer (C50), colon cancer (C18), and rectal cancer (C20). Alcohol RR functions for cancer were obtained from Corrao and colleagues (2004) (For information about the causal relationship between alcohol and cancer, see Baan et al. 2007; International Agency for Research on Cancer 2010.) The alcohol RR for liver cirrhosis (ICD-10 codes: K70 and K74) was obtained from Rehm and colleagues (Rehm et al. 2010*c*). The above-noted RRs were modelled based on drinking status and average daily alcohol consumption among drinkers. The same RRs were used to estimate the AAFs by country, sex, and age for 1990 and for 2010.

Alcohol-drinking statuses and adult (people 15 years of age and older) per capita consumption data for 1990 were obtained from various population surveys (Shield et al. 2013*b*), and the Global Information System on Alcohol and Health (available at: http://apps.who.int/ghodata/?theme=GISAH), respectively. Data on drinking status and adult per capita consumption for 2010 were estimated by projections (performed using regression analyses) using data from years prior to 2010 (Shield et al. 2013*b*). Average daily alcohol consumption was modelled using a gamma distribution (Rehm et al. 2010*b*) and data on per capita consumption for 1990, which was projected to 2010 (Shield et al. 2013*b*). (For more information on the methodology of how alcohol consumption was modelled, see Kehoe et al. 2012; Rehm et al. 2010*b*). This paper presents alcohol consumption data from 2005, the latest year with actual data available.

### Calculating the Alcohol-Attributable Mortality Burden of Injuries

The burden of injury mortality attributable to alcohol consumption was modelled according to methodology outlined by Shield and colleagues (2012), using risk information obtained from a meta-analysis (Taylor et al. 2010) and alcohol consumption data from 1990 and 2010. The risk of an injury caused to the drinker over a year was calculated based on alcohol consumed during normal drinking occasions and alcohol consumed during binge-drinking occasions. Alcohol-attributable injuries caused to nondrinkers also were estimated (Shield et al. 2012).

## Global Consumption of Alcohol

**Figure 1:**
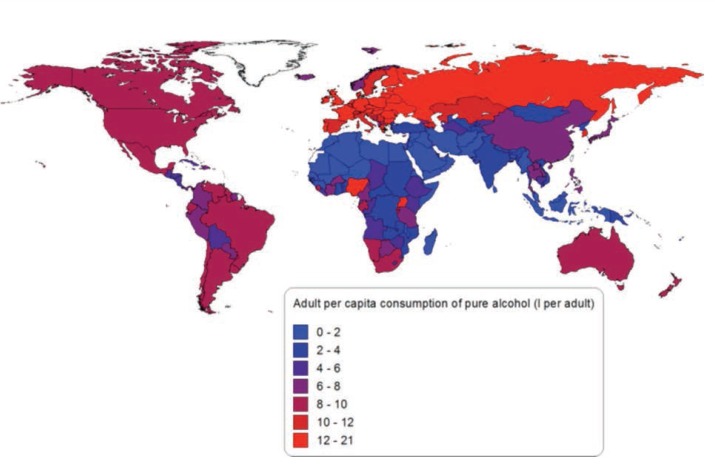
Adult per capita consumption of pure alcohol by country in 2005. NOTE: More detailed information can be obtained from the author.

In 2005 adult per capita consumption of alcohol was 6.1 litres of pure alcohol. Figure 1 shows the adult per capita consumption of alcohol by country. Alcohol consumption per drinker in 2005 was 17.1 litres (9.5 litres per female drinker and 20.5 litres per male drinker). Of all adults, 45.8 percent were lifetime abstainers (55.6 percent of female adults and 36.0 percent of male adults), 13.6 percent were former drinkers (13.1 percent of female adults and 14.1 percent of male adults), and 40.6 percent were current drinkers (31.3 percent of female adults and 49.9 percent of male adults). The global pattern of drinking score (a score from 1 to 5 that measures the harmfulness of alcohol drinking patterns, with 1 being the least harmful and 5 being the most harmful [Rehm et al. 2003*b*]) was 2.6 in 2005 and ranged from 4.9 for Eastern Europe to 1.5 for Western Europe. Thus, alcohol consumption in Eastern Europe can be characterized by frequent heavy alcohol consumption outside of meals and drinking to intoxication.

## Global Alcohol-Attributable Mortality From Cancer

**Figure 2:**
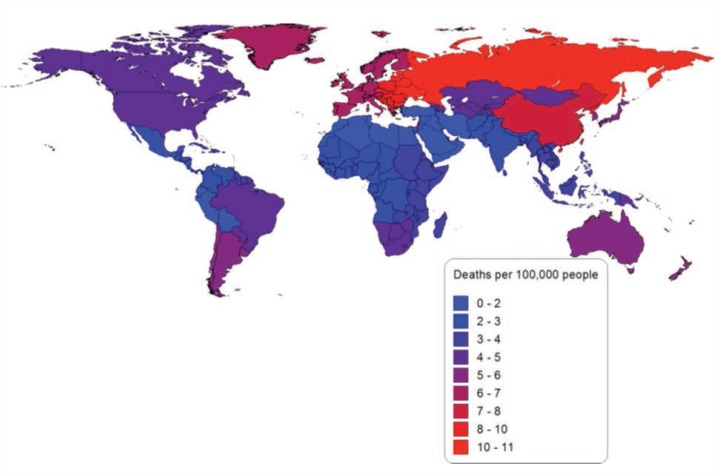
Alcohol-attributable cancer deaths per 100,000 people in 2010 by global-burden-of-disease region. NOTE: More detailed information can be obtained from the author.

In 2010, alcohol-attributable cancer caused 337,400 deaths (91,500 deaths among women and 245,900 deaths among men) and 8,460,000 PYLL (2,143,000 PYLL among women and 6,317,000 PYLL among men). This burden is equal to 4.9 deaths per 100,000 people (2.7 deaths per 100,000 women and 7.1 deaths per 100,000 men) and 122.8 PYLL per 100,000 people (62.8 PYLL per 100,000 women and 181.9 PYLL per 100,000 men). Stated another way, alcohol-attributable cancer was responsible for 4.2 percent of all cancer deaths in 2010 and 4.6 percent of all PYLL caused by cancer. Figure 2 shows the number of alcohol-attributable cancer deaths per 100,000 people by region in 2010. Eastern Europe had the highest burden of mortality and morbidity from alcohol-attributable cancer, with 10.3 deaths and 272.0 PYLL per 100,000 people. North Africa and the Middle East had the lowest mortality burden of alcohol-attributable cancer, with 0.6 deaths and 17.1 PYLL per 100,000 people.

In 1990, alcohol-attributable cancer caused 243,000 deaths worldwide (70,700 deaths among women and 172,300 deaths among men) and 6,405,700 PYLL (1,762,200 PYLL among women and 4,643,500 PYLL among men). This mortality burden is equal to 4.6 deaths per 100,000 people (2.7 deaths per 100,000 women and 6.5 deaths per 100,000 men) and 120.8 PYLL per 100,000 people (67.0 PYLL per 100,000 women and 173.9 PYLL per 100,000 men) caused by alcohol-attributable cancer. From 1990 to 2010 the absolute mortality burden of alcohol-attributable cancer (measured in deaths and PYLL) and the rates of deaths and PYLL per 100,000 people have each increased.

## Global Alcohol-Attributable Mortality From Liver Cirrhosis

**Figure 3:**
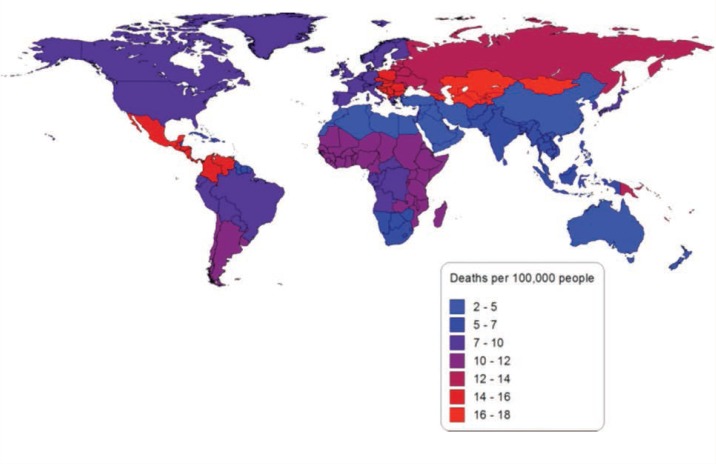
Alcohol-attributable liver cirrhosis deaths per 100,000 people in 2010 by global-burden-of-disease region. NOTE: More detailed information can be obtained from the author.

In 2010, alcohol-attributable liver cirrhosis was responsible for 493,300 deaths worldwide (156,900 deaths among women and 336,400 deaths among men) and 14,327,800 PYLL (4,011,100 PYLL among women and 10,316,800 PYLL among men). This mortality burden is equal to 7.2 deaths per 100,000 people (4.6 deaths per 100,000 women and 9.7 deaths per 100,000 men) and 208.0 PYLL per 100,00 people (117.5 PYLL per 100,000 women and 297.0 PYLL per 100,000 men) caused by alcohol-attributable liver cirrhosis in 2010. Overall, in 2010 alcohol-attributable liver cirrhosis was responsible for 47.9 percent of all liver cirrhosis deaths and 47.1 percent of all liver cirrhosis PYLL. Figure 3 outlines the number of alcohol-attributable liver cirrhosis deaths per 100,000 people by region in 2010, showing strong regional variability.

In 1990, alcohol-attributable liver cirrhosis was responsible for 373,200 deaths worldwide (125,300 deaths among women and 247,900 deaths among men) and 10,906,200 PYLL (3,253,300 PYLL among women and 7,652,900 PYLL among men). That is, 7.0 deaths per 100,000 people (4.8 deaths per 100,000 women and 9.3 deaths per 100,000 men) and 205.7 PYLL per 100,000 people (123.7 PYLL per 100,000 women and 286.6 PYLL per 100,000 men) were caused by liver cirrhosis attributable to alcohol consumption. From 1990 to 2010, the absolute mortality burden of alcohol-attributable liver cirrhosis (measured in deaths and PYLL) and this mortality burden per 100,000 people have each increased (except for women, where alcohol-attributable liver cirrhosis deaths and PYLL per 100,000 decreased slightly).

## Global Alcohol-Attributable Mortality From Injury

**Figure 4:**
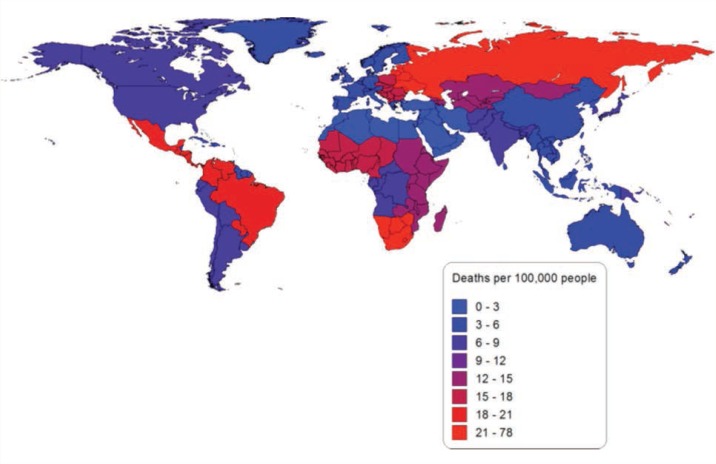
Alcohol-attributable injury deaths per 100,000 people in 2010 by global-burden-of-disease region. NOTE: More detailed information can be obtained from the author.

Globally in 2010, alcohol-attributable injuries were responsible for 669,300 deaths (71,100 deaths among women and 598,200 deaths among men) and 29,110,600 PYLL (3,060,200 PYLL among women and 26,050,400 PYLL among men). This mortality burden is equal to 9.7 deaths per 100,000 people (2.1 deaths per 100,000 women and 17.2 deaths per 100,000 men) and 422.6 PYLL per 100,000 people (89.6 PYLL per 100,000 women and 750.0 PYLL per 100,000 men). Overall, in 2010 alcohol-attributable injuries were responsible for 13.2 percent of all injury deaths and 12.6 percent of all injury PYLL. Figure 4 outlines the number of alcohol-attributable injury deaths per 100,000 people in 2010. Eastern Europe had the greatest mortality burden of alcohol-attributable injuries, with 76.7 deaths and 3,484.7 PYLL per 100,000 people, whereas North Africa and the Middle East had the lowest mortality burden, with 2.0 deaths and 117.2 PYLL per 100,000 people.

In 1990, alcohol-attributable injuries were responsible for 485,100 deaths (54,700 deaths among women and 430,400 deaths among men) and 21,934,800 PYLL (2,409,100 PYLL among women and 19,525,700 PYLL among men), equal to 9.2 deaths (2.1 deaths per 100,000 women and 16.1 deaths per 100,000 men) and 413.8 PYLL per 100,000 people (91.6 PYLL per 100,000 women and 731.3 PYLL per 100,000 men). The absolute number of alcohol-attributable injury deaths and PYLL and the number of alcohol-attributable injury deaths and PYLL per 100,000 people each increased from 1990 to 2010.

Appendix 1 presents the number and percentage of alcohol-attributable cancer, liver cirrhosis, and injury deaths and PYLL by GBD study region for 1990 and 2010. Appendix 2 presents the number of alcohol-attributable cancer, liver cirrhosis, and injury deaths per 100,000 people. Unlike figures 1, 2, and 3, the figures in Appendix 2 use the same scale for each cause of death.

## Global Alcohol-Attributable Cancer, Liver Cirrhosis, and Injury Mortality As Part of Overall Mortality

In 2010, alcohol-attributable cancer, liver cirrhosis, and injury caused 1,500,000 deaths (319,500 deaths among women and 1,180,500 deaths among men). This represents 2.8 percent of all deaths (1.3 percent of all deaths among women and 4.1 percent of all deaths among men), or 21.8 deaths per 100,000 people (9.4 deaths per 100,000 women and 34.0 deaths per 100,000 men). In 1990, alcohol-attributable cancer, liver cirrhosis, and injury caused 1,101,400 deaths (250,800 deaths among women and 850,600 deaths among men), representing 20.8 deaths per 100,000 people (9.5 deaths per 100,000 women and 31.9 deaths per 100,000 men). The table outlines the mortality burden (measured in deaths and PYLL) of alcohol-attributable cancer, liver cirrhosis, and injury for 1990 and 2010 by age and by sex. Compared with the mortality burden in 1990, the absolute number of alcohol-attributable deaths from cancer, liver cirrhosis, and injury in 2010 is higher, and the rate of deaths per 100,000 also increased for men but decreased slightly for women in 2010.

The burden of mortality from alcohol-attributable cancer, liver cirrhosis, and injury led to 51,898,400 PYLL (9,214,300 PYLL among women and 42,684,100 PYLL among men) in 2010 and 39,246,800 PYLL (7,424,600 PYLL among women and 31,822,100 PYLL among men) in 1990. This mortality burden represents 3.0 percent (1.3 percent for women and 4.3 percent for men) of all PYLL in 2010 and 2.0 percent (0.9 percent for women and 2.9 percent for men) of all PYLL in 1990. In 2010, alcohol-attributable cancer, liver cirrhosis, and injury led to 753.4 PYLL per 100,000 people (269.8 PYLL per 100,000 women and 1,228.9 PYLL per 100,000 men) and to 740.4 PYLL per 100,000 people (282.2 PYLL per 100,000 women and 1,191.9 per 100,000 men) in 1990. Again, the overall rates of PYLL per 100,000 people increased, but this effect was attributed to increases for men, coupled with slight decreases for women.

## Measurement Limitations

The methods used to estimate the number of alcohol-attributable cancer, liver cirrhosis, and injury deaths and PYLL have limitations as a result of the available data on mortality and the measurement of alcohol consumption and RRs. Most low- and middle-income countries do not have reliable mortality data and measurement of adult mortality in these countries (through verbal autopsies or surveys) is infrequent. Therefore, estimates of mortality and PYLL have a large degree of uncertainty (Wang et al. 2012). Additionally, for high-income countries, information concerning the cause of death has long been acknowledged as containing inaccuracies (James et al. 1955), and more recent studies have confirmed considerable degrees of error in this information (Nashelsky and Lawrence 2003; Shojania et al. 2003). To adjust for inaccuracies and inconsistencies in mortality data, the 2010 GBD study modelled the number of deaths mathematically (Wang et al. 2012).

Survey data measuring alcohol consumption, patterns of alcohol consumption, and the prevalence of lifetime abstainers, former drinkers, and current drinkers also are susceptible to numerous biases (Shield and Rehm 2012). To correct for the undercoverage that is observed when alcohol consumption is measured by population surveys (as compared with per capita consumption of alcohol), alcohol consumption was modelled by triangulating per capita and survey data (see above). Total alcohol consumption was set to 80 percent of per capita consumption in order to account for alcohol produced and/or sold, but not consumed, and to account for the undercoverage of the alcohol consumption typically observed in studies that calculate the alcohol RRs (Rehm et al. 2010*b*). Additionally, although alcohol was measured using adult per capita consumption and most people 14 years and younger do not consume alcohol or binge regularly, some adolescents 10 to 14 years of age report previously trying alcohol and previously being intoxicated (Windle et al. 2008).

The CRA was based on alcohol RR functions that typically were differentiated by sex and adjusted for age, smoking status, and other potentially confounding factors. The use of adjusted RR functions may introduce bias into the estimated number of deaths and PYLL that would not have occurred if no one had ever consumed alcohol (Flegal et al. 2006; Korn and Graubard 1999; Rockhill and Newman 1998). However, most of the published literature on alcohol-as-a-risk-factor– only reports adjusted RRs, and, thus, the use of unadjusted alcohol RRs for the CRA study would have led to imprecise estimates as a result of leaving out most of the studies. The bias of using adjusted RRs is likely to be small, as most analyses of the estimated RRs show no marked differences after adjustment for the potentially confounding factors and effect measure modifiers. Future CRA studies may require more complex modelling techniques for alcohol if other dimensions of alcohol consumption, such as irregular heavy-drinking occasions, impact RR estimates.

Finally, this analysis did not account for a lag time for the calculation methods used in this paper. This is especially a problem for cancer, which has a lag time of 15 to 20 years (Holmes et al. 2012; Rehm et al. 2007). In other words, the alcohol-attributable deaths and PYLL in 2010 actually are based on consumption patterns from 1990 to 1995, but in this paper were estimated based on consumption in 1990 and 2010. Although liver cirrhosis also is a chronic disease that develops over time like cancer (Rehm et al. 2013*a*), the impact of population-level consumption on liver cirrhosis deaths can be quite abrupt. For example, Gorbachev’s anti-alcohol campaign was reflected in a clear reduction in Russia’s liver cirrhosis mortality (Leon 1997). Likewise, the German seizure of alcohol in France in World War II led to reduced cirrhosis mortality (Zatonski et al. 2010). Most of the effect of alcohol consumption on liver cirrhosis probably is captured within 1 year (Holmes et al. 2012). For injury, with the exception of suicide, there is no noticeable lag time as the risk of injury is associated with blood alcohol content (Taylor and Rehm 2012; see also Cherpitel 2013).

## Implications of Alcohol-Attributable Mortality

In 1990 and in 2010, alcohol consumption had a huge impact on mortality. Regions such as Europe (especially Eastern Europe) and parts of Sub-Saharan Africa (especially south Sub-Saharan Africa) that have a high per capita consumption of alcohol and detrimental drinking patterns are more affected by alcohol consumption compared with other regions. It is important to note that the alcohol-attributable mortality burden is composed of two elements: AAF and the overall mortality burden of the respective disease. Accordingly, the observed overall increase from 1990 to 2010 in alcohol-attributable cancer, liver cirrhosis, and injury deaths and in PYLL can be attributed to two different sources: (1) an increase in the number of cancer, liver cirrhosis, and injury deaths (mainly attributed to increases of these deaths in lowto middle-income countries) (Lozano et al. 2012) and (2) an increase in alcohol consumption in low- to middle-income countries (Shield et al. 2013*b*).

Low- and middle-income countries have higher rates of alcohol-attributable mortality per 100,000 people, even though these countries typically have lower AAF (as their overall burden of mortality is higher). Economic wealth is correlated with overall mortality (Lozano et al. 2012), and, thus, the mortality burden per litre of alcohol consumed is highest in low-income countries, followed by middle-income countries (Rehm et al. 2009; Schmidt et al. 2010). It follows, therefore, that increases in the alcohol-attributable mortality burden in low- and middle-income countries attributed to economic growth may be able to be reduced or controlled for by implementing alcohol control policies such as taxation (Shield et al. 2011; Sornpaisarn et al. 2012*a*, *b*, 2013), bans on advertising, and restrictions on availability (Anderson et al. 2009; World Health Organization 2011*b*) preferably while maintaining the relatively high levels of abstention in these countries.

The typical causes of death associated with alcohol use disorders are liver cirrhosis and injuries, (i.e., exactly the categories described in this article). Liver cirrhosis and injuries, and to a lesser degree cancer, may primarily be responsible for the high proportion of alcohol-attributable mortality explained by alcohol use disorders (Rehm et al. 2013*b*); however, additional research is required to empirically confirm this hypothesis. By increasing the rate of treatment for alcohol use disorders (Rehm et al. 2013*b*), the mortality burden of alcohol-attributable diseases also can be reduced. Recent research has shown that the mortality burden associated with alcohol use disorders, albeit high, has been underestimated (see Harris and Barraclough 1998 for the first meta-analysis; and Callaghan et al. 2012; Campos et al. 2011; Guitart et al. 2011; Hayes et al., 2011; Saieva et al. 2012; Tikkanen et al. 2009 for recent papers that observed a markedly higher mortality risk than in the first meta-analysis).

## Figures and Tables

**Table 1:** Deaths and Years of Life Lost (YLL) From Cancer, Liver Cirrhosis, and Injuries Attributable to Alcohol Consumption in 1990 and 2010

**Year**	**Gender**	**Age (Years)**	**Deaths**	**% Of All Deaths**	**YLL**	**% Of All YLL**
**1990**	Women	0 to 15	4,000	0.1	324,400	0.1
		15 to 34	22,300	1.5	1,349,500	1.5
		35 to 64	128,700	2.9	4,437,000	3.0
		65+	95,800	1.0	1,313,800	1.1
		Total	250,800	1.2	7,424,600	0.9
	Men	0 to 15	6,700	0.1	540,400	0.1
		15 to 34	174,400	8.4	10,547,900	8.4
		35 to 64	502,600	7.4	18,167,100	7.8
		65+	166,800	1.8	2,566,700	2.0
		Total	850,600	3.4	31,822,100	2.9

	**Total**	**Total**	**1,101,400**	**2.4**	**39,246,800**	**2.0**
**2010**	Women	0 to 15	3,800	0.1	313,800	0.1
		15 to 34	28,800	1.7	1,741,700	1.7
		35 to 64	162,000	3.1	5,570,800	3.1
		65+	124,800	0.9	1,587,900	1.1
		Total	319,500	1.3	9,214,300	1.3
	Men	0 to 15	6,100	0.1	492,400	0.1
		15 to 34	214,900	8.5	12,972,300	8.5
		35 to 64	709,200	7.9	25,549,800	8.2
		65+	250,300	1.9	3,669,500	2.2
		Total	1,180,500	4.1	42,684,100	4.3

	**Total**	**Total**	**1,500,000**	**2.8**	**51,898,400**	**3.0**

NOTE: More detailed information can be obtained from the author.
